# The Multidisciplinary Approach to Acute Necrotizing Pancreatitis

**DOI:** 10.3390/jcm14092904

**Published:** 2025-04-23

**Authors:** Dana Ben-Ami Shor, Einat Ritter, Tom Borkovsky, Erwin Santo

**Affiliations:** Department of Gastroenterology, Tel-Aviv Sourasky Medical Center, Affiliated to the Faculty of Medicine, Tel-Aviv University, Tel Aviv 6997801, Israel; einatri@tlvmc.gov.il (E.R.); tombor@tlvmc.gov.il (T.B.); erwins@tlvmc.gov.il (E.S.)

**Keywords:** acute pancreatitis, necrotizing pancreatitis, endoscopic drainage, direct endoscopic necrosectomy

## Abstract

Acute pancreatitis is a common gastrointestinal condition, primarily caused by gallstones and alcohol abuse, with other causes including hypertriglyceridemia, trauma, infections, etc. While most cases are mild and self-limiting, up to 20% of patients develop severe pancreatitis with pancreatic necrosis, increasing the risk of multi-organ failure and mortality. Conservative management involves fluid resuscitation, nutritional support, and antibiotics for infected peripancreatic fluid collections (PFCs). When PFCs are infected or symptoms persist, invasive interventions such as endoscopic ultrasound (EUS)-guided drainage or percutaneous drainage are recommended. Dual modalities (endoscopic and percutaneous drainage) offer better outcomes with fewer complications. Direct endoscopic necrosectomy is considered for patients who do not improve with drainage. A multidisciplinary team, including endoscopists, interventional radiologists, surgeons, and specialists, is essential for optimal management of severe necrotizing pancreatitis.

## 1. Introduction

Acute pancreatitis (AP) is a prevalent digestive system disease and a common cause of hospitalization and illness. The primary causes of AP are gallstone disease and alcohol consumption. Other potential etiologies include metabolic disorders (such as hypertriglyceridemia and hypercalcemia), complications from endoscopic procedures involving the bile ducts or pancreas, certain medications, structural anomalies, trauma, infections, and more [1]. In most cases (80%), acute pancreatitis is a mild condition that resolves spontaneously. However, 10–20% of cases may develop complications, leading to necrosis of the pancreas and/or the surrounding tissues [2].

Pancreatic necrosis typically occurs during the first two weeks of AP. Necrosis may involve only the pancreatic tissue in a minority of cases (less than 5%). In 80% of cases, it affects both the pancreas and the surrounding tissues, while in 20% of cases, only the adjacent tissues are involved [2]. In patients with necrotizing pancreatitis, organ failure is common and can occur even in the absence of infection. The progression of the disease depends on the extent of the necrotic tissue, the degree to which the necrosis spreads to nearby organs, and the development of secondary infections. If the necrotic fluid collection is infected, complications such as multiple organ failure (38%) and the need for mechanical ventilation can occur, leading to elevation in mortality rates up to 30% [3].

This review will discuss severity assessment in acute pancreatitis (AP), the approach to imaging studies in AP, the treatment approach for acute necrotizing pancreatitis (ANP), the strategies for drainage and debridement of pancreatic necrotic collections, and the endoscopic step-up therapy. Lastly, the emerging endoscopic treatment approaches and dedicated devices will be described.

## 2. Severity Assessment in AP

Severity assessment is crucial for guiding management and predicting outcomes. The Revised Atlanta Classification (RAC) [4] is the most widely used system [4], categorizing AP into three severity levels:

1. Mild Acute Pancreatitis (MAP): No organ failure, no local or systemic complications, typically resolves within a few days with supportive care.
2. Moderately Severe Acute Pancreatitis (MSAP): Transient organ failure (resolves within 48 h), local or systemic complications (e.g., peripancreatic fluid collections, necrosis, infection), may require longer hospitalization but generally has a good prognosis.
3. Severe Acute Pancreatitis (SAP): Persistent organ failure (>48 h), affecting at least one of the following: Respiratory (PaO_2_/FiO_2_ < 300)/Cardiovascular (systolic BP < 90 mmHg, unresponsive to fluids)/Renal (creatinine > 1.9 mg/dL). SAP poses high risk of mortality and multi-organ failure.

### 2.1. Scoring Systems for Severity Assessment

Several scoring systems aid in assessing AP severity, including Ranson’s Criteria (assesses severity at admission and at 48 h), the APACHE II Score (used in ICU settings for critically ill patients), the BISAP Score (simple bedside tool for early prediction of severity), and the CT Severity Index (CTSI) (evaluates necrosis and inflammatory response).

### 2.2. Approach to Imaging Studies in AP

According to recent American College of Gastroenterology (ACG) guidelines, routine CT scans at admission are not recommended for assessing acute pancreatitis (AP) severity. CT should be reserved for unclear diagnoses or cases that do not improve within 48–72 h of hospitalization [5]. Pancreatic necrosis appears on contrast-enhanced CT (CECT) as a non-enhancing area, on MRI as a low-signal region compared to healthy tissue [6], and on EUS as a hypoechoic or hyperechoic area within the fluid collection [7].

### 2.3. Classification of Necrotizing Pancreatitis Peripancreatic Collections

The Revised Atlanta Classification of acute pancreatitis from 2012 is an international multidisciplinary classification of the severity of acute pancreatitis. The classification is based on the timing of fluid collection appearance (less vs. more than 4 weeks after the onset of AP) and on the presence of necrosis [8].

1. Acute peripancreatic fluid collection:

A peripancreatic collection that forms in the early stage (up to 4 weeks) after acute pancreatitis, without necrotic components or a defined capsule surrounding it. This process usually resolves spontaneously, and if it persists for more than 4 weeks, it may develop into a pancreatic pseudocyst. In most cases, these collections remain asymptomatic, and treatment is not required [8].

2. Pancreatic pseudocyst:

A fluid collection with a well-defined wall that develops in the late phase (over 4 weeks) following an episode of acute pancreatitis. It is typically located near the pancreas but can occasionally be peripancreatic [9]. On CECT, the pseudocyst displays a characteristic appearance. However, additional imaging, such as endoscopic ultrasound (EUS) or MRI, may be required to rule out any necrotic components within it. Amylase levels are often elevated in fluid aspirated from the pseudocyst during EUS-guided fine-needle aspiration (EUS-FNA). Pancreatic pseudocysts form following injury to the main pancreatic duct or its branches, typically without tissue necrosis [10].

3. Acute necrotic pancreatic collections:

A collection that forms within the first month following an episode of acute pancreatitis, containing both fluid and necrotic tissue. In most cases, the collection involves pancreatic tissue as well as the surrounding peripancreatic area. The main pancreatic duct is often affected due to the progression of necrosis. On contrast-enhanced CT (CECT), the collection typically appears heterogeneous, with areas of non-fluid density and without a well-defined capsule. However, CECT performed within the first four weeks after acute pancreatitis may not reliably differentiate this collection from an acute peripancreatic fluid collection, as pancreatic perfusion impairment occurs gradually. Therefore, in cases with clinical suspicion, follow-up imaging and additional studies, such as MRI and EUS, may be necessary for an accurate diagnosis [11].

4. Walled-off necrosis (WON):

A walled-off necrotic collection in the pancreas or peripancreatic area typically develops more than four weeks after the onset of acute pancreatitis. Differentiating between fluid and necrotic components on CECT can sometimes be challenging, and additional imaging with MRI or EUS may be required [11]. Infection should be suspected if the patient’s clinical condition worsens, if Gram staining or culture of aspirated fluid is positive, or if imaging shows the presence of gas within the collection. Prompt diagnosis is essential to initiate appropriate antibiotic treatment and enable drainage, which may be performed endoscopically, radiologically, or surgically [8].

## 3. The Treatment Approach for Acute Necrotizing Pancreatitis (ANP)

Optimal management of ANP necessitates a multidisciplinary approach, involving experts in pancreaticobiliary surgery, interventional radiology, nutrition, and advanced endoscopy. Conservative management of ANP includes fluid resuscitation, nutritional support, and administration of antibiotics if infection is suspected (Table 1).

### 3.1. Fluid Resuscitation

Early fluid resuscitation in patients with acute pancreatitis is associated with reduced morbidity and mortality [12]. However, studies indicate an increased incidence of respiratory complications, abdominal compartment syndrome, sepsis, and mortality in patients who receive excessive fluid volumes. The European Society of Gastrointestinal Endoscopy (ESGE) guideline, published in 2018, recommends intravenous administration of Ringer’s lactate solution at a rate of 5–10 mL/kg/hour while considering the patient’s respiratory and cardiac status, as well as their underlying conditions, particularly in elderly patients and those with impaired cardiac function. Vital signs, hematocrit, blood urea nitrogen (BUN), creatinine, and sodium levels should be closely monitored [13].

### 3.2. Nutrition

Enteral nutrition is crucial for patients with severe and/or ANP, as it is important for maintaining intestinal mucosal integrity, supporting gastrointestinal motility, and preserving blood flow to the splanchnic circulation. A meta-analysis published in 2008, which included hospitalized patients with acute pancreatitis, found that oral feeding was associated with fewer systemic infections (20% vs. 47%), less multi-organ failure (20% vs. 50%), and lower mortality rates (6% vs. 35%) compared with those receiving parenteral nutrition. Consequently, the American Gastroenterological Association (AGA) recommends initiating oral feeding in patients without nausea, vomiting, signs of impaired intestinal motility, or bowel obstruction [10,14]. If oral feeding is intolerable, nutritional support should begin within 24–72 h.

There is no clear advantage of nasojejunal feeding over nasogastric feeding [7], and using a nasogastric rather than nasojejunal route for delivery of enteral feeding is preferred because of comparable safety and efficacy [5]. For patients who are intolerant to enteral nutrition via a nasal route or who are expected to need prolonged nutritional support (more than 30 days), placement of a feeding tube directly into the stomach or jejunum via endoscopy (percutaneous endoscopic gastrostomy (PEG) or percutaneous endoscopic jejunostomy (PEJ), respectively) should be considered. If enteral nutrition is not feasible due to poor tolerance, bowel obstruction, or impaired motility resulting from the inflammatory response, or if caloric goals are not met through this approach, intravenous feeding (total parenteral nutrition (TPN)) should be considered [15].

### 3.3. Antibiotics

Recent studies have shown that prophylactic antibiotic therapy offers no benefit in reducing morbidity, complications, or mortality in cases of severe acute pancreatitis. In fact, such treatment may even increase the risk of fungal intra-abdominal infections [16].

Infection of the necrotic collection in severe acute pancreatitis can lead to a mortality rate of up to 30% [17]. Hence, prompt diagnosis and intervention are essential. Clinical signs of a septic necrotic process include signs of sepsis, fever > 38 °C, worsening or lack of improvement in the patient’s condition, leukocytosis, bacteremia, and evidence of gas within the collection on imaging studies [18].

If an infection is suspected, antibiotic therapy should be initiated, and drainage of the collection is required. Biopsy or aspiration of the collection fluid under CT guidance can be performed to obtain samples for Gram staining and culture. However, there is a risk of false-positive results and the possibility of secondary infection of a sterile collection after aspiration. Therefore, drainage is not recommended as a routine procedure. In cases of suspected infection, broad-spectrum intravenous antibiotics with good pancreatic penetration (e.g., carbapenems, quinolones, metronidazole, third-generation cephalosporins) should be started. The duration of antibiotic treatment will be determined based on the patient’s clinical condition, hemodynamic parameters, and inflammatory markers at least 48 h after removal of drains [10].

### 3.4. Drainage of Peripancreatic Necrotic Collection

Drainage of a peripancreatic necrotic collection is unnecessary if the patient shows clinical improvement with conservative treatment [19]. Absolute indications for drainage of a peripancreatic necrotic collection include

- Infection of the collection: Confirmed by positive Gram staining or bacterial growth from a culture obtained from the collection fluid.
- High clinical suspicion of infection: Based on clinical deterioration and progression to multi-organ failure, failure to thrive, or evidence of gas within the collection on imaging studies.
- Symptoms resulting from the collection’s pressure on adjacent organs: These may include gastric outlet obstruction or impairment of biliary drainage. Examples of symptoms indicating the need for drainage include nausea, vomiting, early satiety, abdominal fullness, weight loss, and persistent abdominal pain [20].

## 4. The Approach to Drainage of Pancreatic Necrotic Collections

Endoscopic transmural drainage for necrotic pancreatic collections was initially reported in 1996 [21] and has since evolved with the development of various techniques. Such drainage is viable when the proximity of the collections to the gastric or duodenal wall permits safe access. Collections near the pancreatic head are generally drained through the duodenal wall, while those in the pancreatic body or tail are approached via the gastric wall.

**Figure 1:**
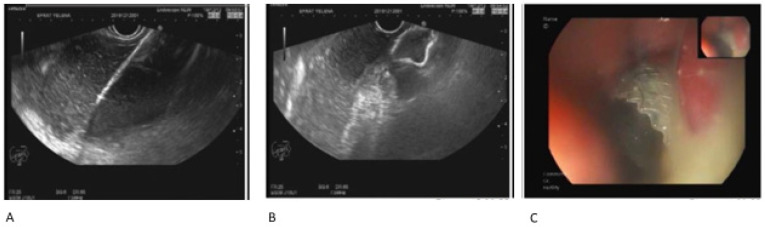
Endoscopic drainage of a pseudocyst using a metal stent. (**A**,**B**): EUS-guided endoscopic stent insertion through the gastric wall into the pancreatic collection; (**C**): Endoscopic view of the stent. Source: Department of Gastroenterology, Tel-Aviv Sourasky Medical Center.

Historically, drainage was performed “blindly” by targeting bulging collections protruding into the gastric or duodenal lumen. Today, however, EUS-guided drainage has become the standard approach [15,22] (Figure 1). EUS-guided drainage has significantly higher success rates compared to “blind” drainage, with success rates reported to be 94–100% vs. 33–72%. The primary advantage of EUS guidance lies in its ability to treat collections that do not protrude into the gastric or duodenal lumen. Additionally, the use of Doppler imaging during needle puncture allows for the assessment of blood flow, minimizing the risk of passing through blood vessels near the collection site. However, it is important to note that postponing the drainage of an infected collection for one to four weeks is preferred and results in fewer necrosectomies and other interventions (radiological, surgical, and endoscopic), with no difference in complications or mortality [23]. Obviously, this approach relates to patients who have mild symptoms and are in a stable condition.

**Figure 2:**
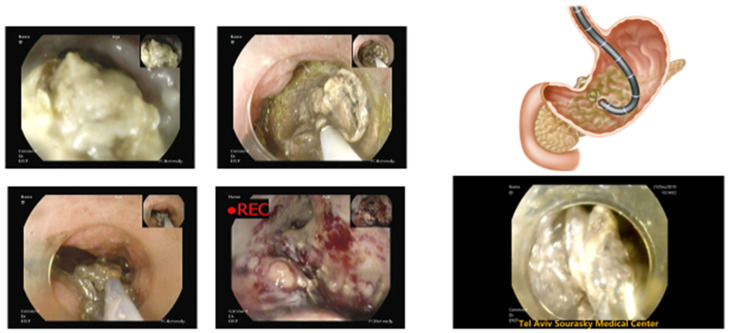
Direct endoscopic necrosectomy. Source: Department of Gastroenterology, Tel-Aviv Sourasky Medical Center.

Previously, following the puncture of the necrotic collection, balloon inflation was used to create a fistula between the collection and the gastrointestinal lumen. Plastic double-pigtail stents were then placed to maintain the fistula, facilitating fluid drainage from the collection into the stomach or duodenum [24]. Subsequently, the introduction of self-expandable metal stents (SEMS) revolutionized the approach: compared to plastic stents, SEMS offer a larger diameter, enabling efficient drainage of necrotic material and improving overall procedural outcomes. The advent of lumen-apposing metal stents (LAMS) has revolutionized the endoscopic treatment of necrotizing pancreatitis. These newer stents are shorter in length and feature a wide diameter, allowing passage of an endoscope through them. LAMS offer significant advantages, including ease of placement and the ability to perform direct endoscopic necrosectomy (DEN) for debridement of the collection when necessary [13,25,26] (Figure 2).

EUS-guided placement of LAMS has a high technical (92–100%) and clinical success rate [27,28]. In certain cases, multiple transmural access points to the necrotic cavity may be necessary—a technique known as the multiple transluminal gateway technique. According to the 2018 European Society of Gastrointestinal Endoscopy (ESGE) guidelines, this approach should be considered in patients with collections larger than 12 cm, multiple collections, or when there is an inadequate response to single-tract transmural drainage [13]. Additionally, the ESGE recommends removing the LAMS within four weeks of placement to reduce potential complications.

## 5. Percutaneous Catheter Drainage (PCD)

Percutaneous drainage guided by CT or ultrasound is often necessary during the early stages of inflammation, particularly when infection is suspected. This approach is also indicated for fluid collections that are distant from the duodenal or gastric wall, often extending toward the paracolic gutters. The dual-modality approach, which integrates endoscopic and percutaneous drainage, has demonstrated improved success rates. Two retrospective studies evaluating drainage strategies for necrotic collections reported success rates ranging from 35% to 51% [29,30]. Percutaneous drain enhances the efficacy of endoscopic drainage by enabling a combined protocol of irrigation within the collection cavity through the percutaneous drain. The advantages of combining endoscopic and percutaneous drainage, compared to percutaneous drainage alone, include a reduced incidence of pancreatic-cutaneous fistulas, decreased need for repeated drainages, fewer endoscopic procedures, reduced need for imaging studies, shorter hospitalization duration, and a lower rate of hemorrhagic complications [31].

## 6. Necrotic Peripancreatic Collection Debridement

The combination of endoscopic and percutaneous drainage provides effective treatment for most peripancreatic collections. However, in cases where clinical improvement is insufficient, progressing to necrotic tissue debridement may be warranted. The traditional approach to necrotizing pancreatitis debridement is open surgical intervention, aimed at achieving complete removal of the infected necrotic tissue [32]. This approach is associated with a high complication rate (34–95%) and a mortality rate of 11–39%. Endoscopic debridement, however, offers a minimally invasive alternative, with a reported success rate of 82–100% and significantly lower complication rates, including bleeding in 1%, perforation in 5%, and mortality in 10% [20,33]. Additionally, the length of hospitalization following endoscopic debridement is shorter compared to surgical debridement [34].

The addition of irrigation during endoscopic stent placement has demonstrated promising results in managing infected pancreatic collections effectively while minimizing complications associated with infection persistence [35]. In our personal experience, daily irrigation through a nasocystic drain with saline solution 2000 cc per day for 7 days resulted in resolution of the infection without the need to perform necrosectomy in a high proportion of cases (unpublished data).

## 7. Predictors for Necrosectomy Debridement

Several factors influence the decision to perform debridement and the choice of technique. The predictors associated with the need for surgical intervention following endoscopic treatment include the size of the necrotic collection (>18 cm), extension to the paracolic gutter, and a history of diabetes [36]. Additional factors impacting the clinical course include the extent of necrotic tissue within the collection, prolonged organ failure, and elevated CRP levels.

Comparisons between direct endoscopic necrosectomy (DEN) and other debridement methods have been explored in various studies:

1. DEN vs. video-assisted retroperitoneal debridement (VARD):

Lower incidence of multi-organ failure in the endoscopic approach (0% vs. 50%) has been noted in a study of 22 patients, with no significant difference in mortality. DEN showed reduced fistula formation and shorter hospitalization [29,37].

2. DEN vs. percutaneous drainage:

The endoscopic approach demonstrated higher clinical resolution rates (92% vs. 64%) and reduced hospitalization duration [38].

3. DEN vs. open surgical debridement:

Comparable clinical success rates but a lower complication rate and shorter hospital stays have been noted with DEN. However, no mortality benefit was observed with the endoscopic approach [10].

## 8. Debridement Optimization

The use of hydrogen peroxide irrigation during endoscopic procedures has been shown to enhance debridement by facilitating the removal of necrotic tissue. Hydrogen peroxide facilitates the debridement of necrotic tissue and has been shown to improve success rates, achieving 94% success with H_2_O_2_ irrigation compared to 79% without its use (*p* < 0.02), without increasing adverse effects [39]. In cases where infection of the collection cavity is suspected, some protocols include local treatment with gentamicin-based antibiotic irrigation [40]. Additionally, discontinuing proton pump inhibitors (PPIs) is recommended, as acidic gastric pH may accelerate the liquefaction of necrotic tissue. A retrospective analysis investigating the association between PPI therapy and the resolution of necrotic collections found that patients receiving PPI therapy required more frequent debridement [41].

## 9. Endoscopic Step-Up Therapy

In cases of infected necrotic processes or symptomatic conditions, the initial consideration should be whether the process is organized and accessible for transmural endoscopic drainage. If endoscopic drainage is feasible, the first preference is EUS-guided drainage using a LAMS. According to the recommendations of the American Gastroenterology Association (AGA) as well as the European Society of Gastrointestinal Endoscopy (ESGE) [10,13], the stent should be removed within four weeks of placement. This recommendation is based on evidence of an increased risk of bleeding complications in patients where the LAMS was left in place for a longer duration [22].

With regard to collections that are not endoscopically accessible or extending toward the gutters, the preferred approach is percutaneous drainage [36,40]. A controlled clinical study from 2010 compared the endoscopic step-up approach to primary surgical debridement via an open approach. The study demonstrated advantages for the step-up approach in terms of severe complications, mortality, the development of new-onset diabetes, and the need for pancreatic enzyme replacement therapy during a six-month follow-up. These benefits were sustained during long-term follow-up of 86 months for the same patient cohort [30,42].

A prospective study enrolled 66 patients with infected necrotic pancreatic collections. Patients were randomized to either minimally invasive surgery or a step-up endoscopic approach, with or without endoscopic debridement. The primary outcome was mortality or major complications within six months. The endoscopic step-up group had a significantly lower rate of the primary outcome (11.8% vs. 40.6% in the surgical group, *p* = 0.007). However, there was no significant difference in overall mortality between the two groups [22]. In another study with a five-year follow-up, patients treated with the endoscopic step-up approach were compared to those treated with the surgical approach.

While there was no significant difference in overall mortality or major complications, the endoscopic approach showed a significant advantage in terms of cutaneous pancreatic fistula rates (8% vs. 34%) and required fewer additional interventions at the six-month follow-up (7% vs. 24%) [43]. Based on these and other studies, the endoscopic step-up approach is emerging as a leading paradigm in the management of patients with infected necrotizing pancreatitis.

## 10. Timing of Direct Endoscopic Necrosectomy (DEN) of Walled-Off Necrosis (WON)

Historically, after initial drainage of infected necrotizing pancreatitis, if no clinical improvement is achieved, direct endoscopic necrosectomy is performed in a step-up approach in order to facilitate early resolution of walled-off necrosis. In the past, DEN was associated with potentially higher rates of lethal adverse events, such as bleeding, perforation, and peritonitis. However, due to advancement in endoscopic techniques and expertise, the safety of DEN has improved. As a result, there has been a debate regarding the timing of DEN after initial drainage. Two recent RCTs challenged the conventional approach and hypothesized that immediate DEN after initial drainage will result in shorter time to resolution of WON, with no difference in adverse events. In the DESTIN trial [44] patients undergoing immediate DEN after initial drainage had fewer overall interventions (median of 1 vs. 2, *p* = 0.003), and shorter hospital stay (9 days vs. 19 days, p=0.048) than the step-up approach. The WONDER-01 trial [45] additionally showed that time for recovery was shorter in the immediate DEN group (29 days vs. 44 days, *p* = 0.009). In both trials, there was no difference in adverse events or mortality between the two groups.

This step-up multidisciplinary approach was reported in a large, retrospective, observational study. In total, 83 patients with necrotizing pancreatitis were managed at a tertiary referral center between 2009 and 2014. The step-up approach prioritized endoscopic transluminal drainage (ETD) and endoscopic transluminal necrosectomy (ETN), with adjunctive percutaneous catheter drainage (PCD) when necessary. Treatment success was achieved in 73% of patients, with 86% success in those treated endoscopically alone. The mean number of endoscopic interventions was 3.8, and only 4% of patients required open surgical necrosectomy. Adverse events occurred in 13% of patients, including bleeding (11%), perforation (1%), and one fatal air embolism. Mortality was 7%, primarily due to sepsis. The study highlights the effectiveness of a minimally invasive step-up approach, with fewer complications and a reduced need for surgery compared to traditional open necrosectomy [46]. Furthermore, clinical outcomes were compared between the step-up approach and open necrosectomy in a prospective cohort study involving 99 consecutive patients with necrotizing pancreatitis. Patients in the step-up approach group had significantly lower morbidity and pancreatic insufficiency rates, while hospital stay duration and mortality rates were comparable between the two groups. If an abdominal emergency develops, such as organ perforation, abdominal compartment syndrome, or massive bleeding, surgical treatment should be considered. In all other cases, less aggressive treatment can be initially applied [47].

## 11. Emerging and Future Innovations in DEN

One of the main limitations of endoscopic necrosectomy is the lack of dedicated, effective instruments for efficiently removing necrotic tissue. Currently, various tools originally designed for other procedures are employed for this purpose. Instruments such as lithotripsy baskets, grasping forceps, retrieval nets, and polypectomy snares are often used, but they typically lack the necessary grip to secure tissue effectively. This makes the procedure cumbersome, time-consuming, and often only marginally successful, as only small chunks of necrosis can be removed with each pass. Additionally, the opening of these devices in necrotic areas is largely uncontrolled, both visually and in terms of the amount of tissue captured. While suction can assist in removing tissue, it frequently causes clogging of the endoscope’s working channel, further complicating the procedure.

At present, there is initial experience with a dedicated automatic mechanical endoscopic resection system, the EndoRotor (Interscope, Inc., Whitinsville, MA, USA) [48]. The EndoRotor is a specially designed endoscopic tool that enables effective and controlled debridement of necrotic tissue with minimal disruption to surrounding structures. It can suction, cut, and remove small tissue fragments through a catheter, which consists of a fixed outer cannula and a hollow inner cannula. A motorized, rotating cutting tool, driven by an electronically controlled console, performs tissue resection and rotates at speeds of either 1000 or 1700 revolutions per minute. Necrotic tissue is sucked into the catheter using negative pressure and cut by the rotating blade of the inner cannula. Both the cutting tool and suction are controlled by the endoscopist using two separate foot pedals [48]. A recent systematic review and meta-analysis has suggested that the EndoRotor is a safe and effective tool for treating pancreatic necrosis, potentially reducing the number of debridement sessions compared to conventional instruments [49].

Additional experimental innovations that could potentially advance the boundaries of direct endoscopic necrosectomy (DEN) have recently been reported. Preliminary results suggest the feasible and safe use of a novel dedicated device, Necrolit (Meditalia, Palermo, Italy) [50]. Necrolit is a new medical device designed specifically for use in DEN. The 3.1 mm double-lumen multi-action catheter features both a 25 mm nitinol basket and a 25 mm stainless-steel ultra-stiff loop. These components are embedded in an “all-in-one” device, controlled by a three-ring hub for maneuvering the loop and a stick that moves the handle to maneuver the basket. The use of the loop or basket can be alternated without the need to exchange the device. The basket is primarily used for grasping and removing muddy necrotic material, while the ultra-stiff loop is ideal for grasping the thicker portions of necrosis, which are ensnared and excised with a monofilament oval wire.

Hydrojet-assisted necrosectomy uses high-pressure water jets to debride necrotic tissue while preserving viable pancreatic tissue, potentially reducing mechanical trauma compared to traditional forceps or snares. The waterjet necrosectomy device (WAND) was designed to deliver a controllable waterjet force capable of safely fragmenting necrosis while minimizing trauma to healthy, non-target tissue. Early investigations, including benchtop, ex vivo, and in vivo (porcine) testing, demonstrated that the WAND prototype effectively irrigates and fragments necrotic debris ex vivo while avoiding damage to the surrounding healthy tissue [51].

An additional creative direction would be a robotic-assisted endoscopic system to automate and optimize necrosectomy, as well as AI-powered computer vision algorithms that may help identify necrotic vs. viable tissue during procedures. As these innovative strategies continue to evolve, they hold promise for reducing the morbidity and mortality associated with ANP.

## 12. Summary

Necrotizing pancreatitis is a severe form of acute pancreatitis associated with high mortality and morbidity, and often, a prolonged hospital stay. A staged, minimally invasive “step-up” approach is effective in treating necrotizing pancreatitis. This strategy begins with maximal supportive therapy followed by percutaneous or endoscopic drainage of infected pancreatic collections. If necessary, minimally invasive surgical debridement is performed, delaying surgery until the necrotic tissue becomes encapsulated.

Implementing this multidisciplinary approach involves the collaboration of gastroenterologists, interventional radiologists, pancreatic surgeons, infectious disease specialists, nutritionists, and critical care experts. Such collaboration has been shown to reduce mortality rates and result in fewer major complications compared to traditional methods. Therefore, a coordinated, team-based strategy is essential for optimizing the management and outcomes of patients with necrotizing pancreatitis.

## Figures and Tables

**Table 1:** Comprehensive treatment approach for acute necrotizing pancreatitis.

Treatment Approach	Key Points
Fluid Resuscitation	- Early resuscitation reduces morbidity/mortality, but excess fluids increase risks (respiratory issues, sepsis). - ESGE recommends IV Ringer’s lactate (5–10 mL/kg/hour). - Monitor vitals, hematocrit, BUN, creatinine, sodium.
Nutrition	- Enteral nutrition preferred for severe cases (preserves gut integrity, reduces infections and organ failure). - Oral feeding recommended unless not tolerated. - PEG/PEJ for prolonged support (>30 days). - TPN if enteral feeding is not feasible.
Antibiotics	- No benefit of prophylactic antibiotics; may increase fungal infections. - Suspected infection requires broad-spectrum IV antibiotics (carbapenems, quinolones, metronidazole, cephalosporins). - Consider drainage if infection confirmed.
Drainage of Necrotic Collection	- Only necessary if infection is confirmed, high clinical suspicion, or symptomatic (e.g., gastric outlet obstruction). - EUS-guided transmural drainage preferred over blind techniques. - LAMS improve outcomes. - Percutaneous drainage for distant collections or when endoscopic approach is not feasible.
Necrotic Tissue Debridement	- Predictors for necrosectomy: Large collections (>18 cm), paracolic gutter extension, diabetes. - Hydrogen peroxide irrigation improves success. - Avoid PPIs, as they may slow necrosis resolution.

Legend: Blood urea nitrogen (BUN); endoscopic ultrasound (EUS); European Society of Gastrointestinal Endoscopy (ESGE); intravenous (IV); lumen-apposing metal stents (LAMS); percutaneous endoscopic gastrostomy (PEG); percutaneous endoscopic jejunostomy (PEJ); proton pump inhibitor (PPI); total parenteral nutrition (TPN).
